# A Guide Inside Electrochemiluminescent Microscopy Mechanisms for Analytical Performance Improvement

**DOI:** 10.1021/acs.analchem.1c05065

**Published:** 2021-12-15

**Authors:** Sara Rebeccani, Alessandra Zanut, Claudio Ignazio Santo, Giovanni Valenti, Francesco Paolucci

**Affiliations:** † Department of Chemistry “Giacomo Ciamician”, University of Bologna, Bologna 40127, Italy; ‡ Tandon School of Engineering, New York University, Brooklyn, New York 11201, United States

ECL is luminescence generated by electrochemical reactions and for this reason it possesses better spatiotemporal control and low background in comparison with photoluminescence or other optical methods that rely on external light illumination.^1−3^ In the last 20 years, ECL has proved to be a versatile and powerful analytical technique in different fields, ranging from fundamental research to commercial clinical and biological applications.^4,5^ The main reason behind its success is that ECL offers remarkable advantages in comparison to other transduction methods: high sensitivity, an extremely wide dynamic range, very low background signal, good temporal and spatial control, and insensitivity to matrix effects.^6,7^ Thanks to its simplified optical setup, ECL has been implemented as a powerful imaging technique to visualize electrochemical objects and entities bringing important insight in the ECL mechanism generation. Here we aim to incorporate all the work done in the field of ECL imaging mainly in the last 3 years with a particular focus on ECL generation mechanisms and their applications.

ECL essentially implies the production of light by an excited luminophore species that is generated at the electrode surface, through the exergonic electron transfer (ET) reactions among the electrogenerated species.^8^ Differently from similar chemical systems (i.e., chemiluminescence) where the light is produced in the bulk by mixing the reactive species, ECL is initiated by applying the electrode potential and the reagents are produced in situ electrochemically and thus is independent of the fluid flow.^9^

ECL processes have been established for several different molecules and nanosystems according to two main mechanisms, annihilation or by the use of a coreactant.^10^ The first observations of light emission during electrolysis were published in the 1920s;^11^ however, a turning point for ECL application was the first example in 1970s of ECL derived from electrogenerated species of the [Ru(bpy)_3_]^2+^ complex followed by the discovery of ECL emission in aqueous media with tri-*n*-propylamine (TPrA), which is the most efficient coreactant used nowadays for (bio)analytical applications.^9,12^

A coreactant is a chemical species that undergoes an electrochemical oxidation or reduction at the electrode surface producing very reactive intermediates capable of reacting with the luminophore to generate the excited state.^8,11^ Typical coreactants are amines, such as TPrA and 2-(dibutylamino)ethanol (DBAE), peroxydisulfate, NADH, or H_2_O_2_, and based on their features, they may generate ECL through the so-called “oxidative-reduction” or “reductive-oxidation” mechanisms.^13−17^

The main mechanism for the generation of ECL with the couple [Ru(bpy)_3_]^2+^/TPrA is called “homogenous ECL”; it exploits the “oxidative-reduction” scheme and involves the direct oxidation of both the luminophores and the coreactant at the electrode surface followed by the deprotonation reaction of the oxidized coreactant that generates TPrA^•^. The so generated TPrA^•^ and [Ru(bpy)_3_]^3+^ react in the diffusion layer and generate excited state [Ru(bpy)_3_]^2+^*, which decays by emitting light (Figure 1A).

**Figure 1:**
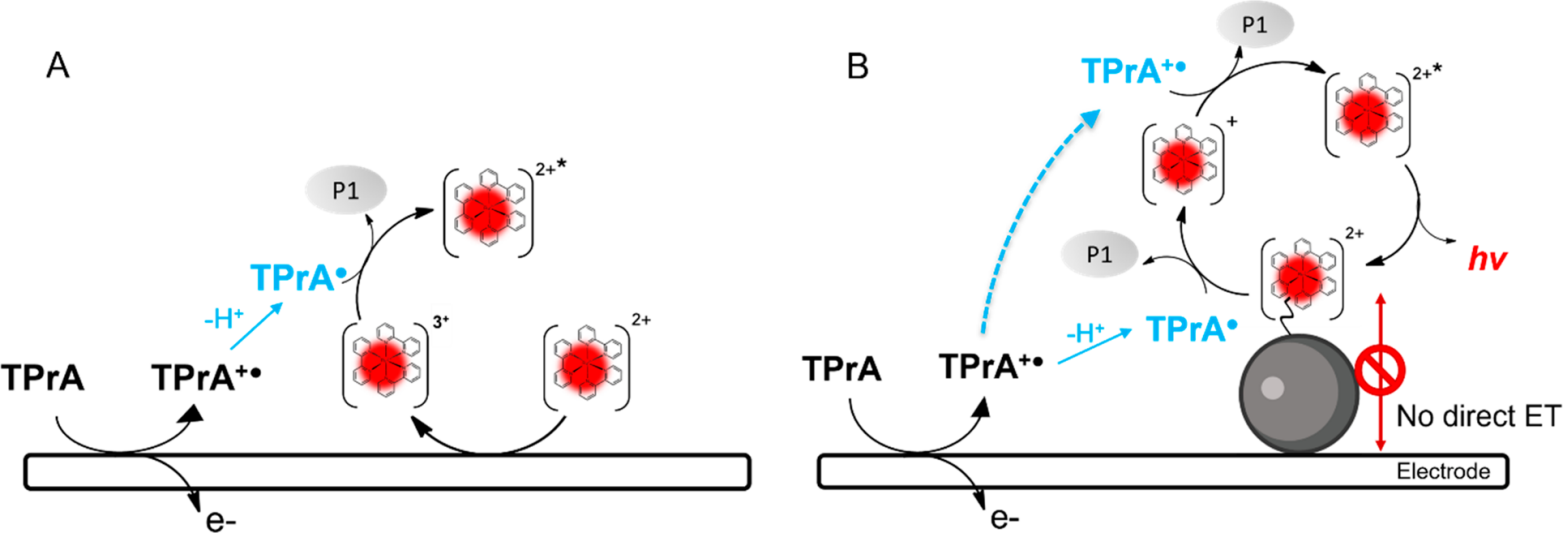
Schematic representation of electrochemiluminescent mechanism: (A) homogeneous where both coreactant and luminophore can be oxidized at the electrode surface and (B) heterogeneous where only coreactant is oxidized.

In most of the analytical methods, however, the luminophore is constrained in proximity to the electrode because it is attached to a sensing element (i.e., antibody, bead, DNA probe, etc.) and thus not free to diffuse to the electrode.^18^ In this case, ECL emission is therefore triggered exclusively by the radicals obtained by the anodic oxidation of TPrA, i.e., TPrA^•+^ and TPrA^•^ that, diffusing from the electrode surface, would react with the immobilized luminophore generating the excited state (Figure 1B).^11^ According to this path, “heterogenous ECL” light is generated by the participation of both radicals continuously flowing across the region where luminophores are located to sustain the production of the excited state and a spatial distribution of ECL efficiency may be expected, more efficient where TPrA^•+^ and TPrA^•^ coexist and where their concentrations are simultaneously the highest. Recently, inspired by the same mechanistic leading guide, a new family of coreactants/additives has been proposed that enhances the ECL signal in a commercial immunoassay system for the quantification of several biomarkers.^4^

As mentioned before, an important breakthrough to elucidate the ECL mechanism was the introduction of advanced optical readouts for imaging applications. An ECL microscopy setup includes a bright field microscope, an electrochemistry module (including an electrochemical cell and a potential generator), and a charge coupled device (CCD) or spectrometer acquiring the image or spectrum. For a high spatial resolution image, an objective with a high numerical aperture value and an electron multiplying CCD (EMCCD) are needed to increase the collection efficiency.^19^ To minimize the luminescence loss during the transmission, the distance between the sample and CCD should be as short as possible. Moreover, the mirrors or filters in the transmission pathway are recommended to be removed except for special applications.

ECL imaging has been successfully used to analyze species over the electrode surface, in which spatially and temporally resolved electrochemical signals were well separated from surrounding information.^6,20^ These important breakthroughs allowed the parallel measurement of samples or the observation of electrochemical and/or biological events at the regions of interest. Moreover, the luminescence process is induced by an electrochemical stimulus, thus, in contrast with other optical microscopy techniques, no optical excitation is required. The absence of excitation light minimizes the background from scattered light or sample autofluorescence that is important especially for biological applications. Altogether, ECL imaging possesses all the advantages of the electrochemical sensitivity together with the spatial resolution provided by its optical readout.

In the years, a significant number of works has been carried out to create electrode architectures and to design new ECL probes with the aim to improve detection strategies as well as the quality of the ECL image.^21−23^ Because of this, ECL imaging is nowadays an important tool for performing multiplex bioassays, characterizing the cellular behavior of single cells and the electrochemical features of single particles.^24−26^ Below we will describe how ECL imaging has allowed to obtain a deeper insight in the mechanisms to generate ECL and how ECL imaging has been applied to visualize different objects, cells, and subcellular components at the electrode surface, sometimes with unprecedented resolution.

## ECL Imaging

### Homogeneous Mechanism Improvement and ECL Enhancement

The ECL direct oxidative mechanism is the first, most intuitive, and easy applicable mechanism discovered in the field of aqueous ECL involving a coreactant.^11,27^ Recently, the deep understanding of this apparently simple ECL generation mechanism allowed some very innovative applications, mainly in the bioanalytical field^24,28^ as well as in the wider field of sensors and the nanoworld.^29^ New strategies and new materials^30,31^ were discovered and investigated to increase ECL sensitivity. In particular, the combination with microscopy gave a great boost in the use of ECL as an analytical technique.^3^ New insights into the direct oxidative mechanism were further enlightened by ECL microscopy and exploited for more sensitive applications, underling the amazing features of this promising technique.

Adamson et al. clearly showed how mechanistic results can be very useful for the development of more sensitive analytical applications such as qualitative screening of drug types detection.^32^ In their work, they observed different emissions at working and counter electrodes, exploring the potential-dependent multicolor coreactant ECL. In the system, different luminophores were simultaneously present together with TPrA. The interactions mechanisms of different metal-complex luminophores (also without TPrA) were elucidated, underlying the dependence of the different colors emission not only on the type of luminophore but also on the electrode material and applied potential. These experiments were however performed in acetonitrile, and organic solvents are not suitable for environmental and medical applications and, more in general, in the commercial field.^33^ In fact, in the last years, a consistent part of research involving ECL microscopy has been conducted in aqueous systems. Since ECL is essentially a surface confined technique with high spatial and temporal resolution, the roles of the coreactant and luminophore are fundamental; thus, insights on the mechanistic pathway to generate signals are of great importance to improve ECL applications. In particular, coreactant lifetimes of the radical cation and luminophore in their oxidized form represent the main rate-limiting steps in the ECL signal generation affecting especially the direct oxidative route in which both the luminophore and coreactant are free in solution.

The reagents lifetime limits the ECL vertical resolution, and mechanistic insights were successfully investigated by Voci et al. exploiting the reduced volume of a nanochannel device.^34^ ECL was generated applying 2 V at the electrode at the bottom of the nanochannel and 0 V potential at the top electrode to avoid the reduction of luminophore [Ru(bpy)_3_]^2+^ and consequently the annihilation pathway.^2^ Luminescence was observed only at the level of access holes of the nanochannel, and this limitation is due to the diffusion of oxidized [Ru(bpy)_3_]^3+^ and the lifetime of the coreactant TPrA, as previously anticipated. The Ru[(bpy)_3_]^2+^* diffusion distance into the nanochannel was <1 μm due to the lifetime of the TPrA radical cation (TPrA^•+^) before deprotonation, which has a decay rate of 3500 s^–1^.^5,17,18^ Finally, the light emission was excluded from the strongly confined volume of a nanochannel because of coreactant depletion which is responsible for the different spatial ECL emission.

Confined geometries, like nanochannels, enhance and restrict the ECL emission region that can be easily spatially resolved. For these reasons, nanochannels or microtubes were largely used for the investigation of the direct oxidative mechanistic route. Important insights were deciphered by Bin Su and co-workers exploiting ultrahigh-density gold microwells,^35^ microtubes,^36^ and an ensemble of microtube electrodes.^37^ They derived information on the ECL emitting layer, a very important parameter for ECL sensitivity modulation that, on one hand, when it is thin, allows the visualization of nanometric objects confined on the electrode with high throughput, while on the other hand, when thicker, it may improve the ECL signal thus visualizing entire and micrometric objects. Surely, several factors and in particular the experimental conditions influence the ECL emission layer thickness (TEL) and intensity: (i) the electrode material,^22^ (ii) the coreactant,^14,15^ (iii) the applied voltage, and (iv) the acquisition time.^38^

The fabrication of a gold coated polydimethylsiloxane ultrahigh-density gold microwell electrodes (UMEA) was very useful to study the ECL reaction process at the single microwell level and the spatial resolution of the emission when combined with ECL imaging.^35^ The resulting ring-shaped ECL emission from [Ru(bpy)_3_]^2+^ and TPrA is proof of the enhanced and restricted ECL intensity in confined geometries, allowing a spatial resolution of ECL rings. The ECL emission intensity results are stronger inside the well compared to the top surface, mainly at the junction between the sidewall and the bottom of a single well. The [Ru(bpy)_3_]^2+^ distribution is concentrated at the bottom of UMEA as it is visible from COMSOL finite element simulation. The behavior and ECL enhanced emission in confined space arises from the superposition of radial and longitudinal diffusion fields. It is possible to decipher the type of ECL mechanism involved in the emission changing the concentration of [Ru(bpy)_3_]^2+^ and the exposure time for acquisition of images with a CCD camera: (i) at low concentration, direct oxidative coreactant mechanism is involved; (ii) at high concentration and increasing the acquisition time, the ECL emission shape changes from a ring to a spot, a symptom of the involvement of the catalytic route. In conclusion, this study showed that at low [Ru(bpy)_3_]^2+^ concentration, the ECL emission is limited by the short lifetime of TPrA radicals, while at high [Ru(bpy)_3_]^2+^ concentration, the diffusion profile of [Ru(bpy)_3_]^3+^ is the limiting factor and the thicker TEL is due to the higher lifetime of Ru(bpy)_3_^3+^. It can catalyze the oxidation of TPrA farther from the electrode if the acquisition time of CCD camera is high enough to allow [Ru(bpy)_3_]^3+^ diffusion. Short TPrA radical lifetimes do not limit the TEL anymore.^35^

Another study on microtube electrodes (MTE) confirmed the behavior while giving more information about the correlation between TEL, type of coreactant (TPrA or DBAE) involved in the ECL emission, and [Ru(bpy)_3_]^2+^ concentration.^36^ The TEL, at a high luminophore concentration, has a thickness between 3.1 and 4.5 μm, ever larger than expected from computational studies.^39^ However, using DBAE as a coreactant, the shape of ECL emission did not change with [Ru(bpy)_3_]^2+^ concentration because of the reduced lifetime of DBAE radicals, which is much shorter than for TPrA, and thus the [Ru(bpy)_3_]^3+^ concentration increase has no effect on the TEL.^36^

Nonconductive channels are also very important in ECL microscopy for visualization of the cell and their cell-matrix adhesion structures like spikes, podosomes, lamellae, ruffles, and focal or fibrillar adhesion, which are important markers for changes in cells activities and allowing a dynamic monitoring. Exploiting the common direct oxidative mechanism is possible to visualize the cell or their structures exploiting a new “negative ECL” strategy: the object hinders the ECL reagents diffusion and consequently the emission, visualizing a spot surrounded by ECL emission. One example involves the use of PC12 target cells cultured on the surface of indium tin oxide (ITO) electrode modified with a silica nanochannel membrane (SNM), which has high sensitivity and nonconductivity, thus reducing the electrical and chemical perturbation of cells.^7^ In the presence of TPrA and [Ru(bpy)_3_]^2+^, these nanochannels allow the “negative” visualization of cell-matrix adhesion sites for two main reasons: (i) their negative surface charge attracts the positively charged luminophore; and (ii) they increase the visual contrast increasing the distance/volume between the basal cell membrane and the underlying ITO electrode surface. Negative ECL or “shadow ECL” do not require the labeling by a specific dye, avoiding cells modification due to surface functionalization and/or pretreatment. This labeling-free approach combined with ECL microscopy was exploited also for the visualization of small subcellular organelles and living mitochondria.^40^ This technique has a better optical contrast compared to fluorescence obtained using a specific dye. It can visualize mitochondria with an area between 0.5 and 6 μm^2^ without altering their metabolic redox status. The involved direct oxidative mechanistic route was deciphered by observation of an ECL-emitting layer confined to the micrometric distance from the electrode surface and by monitoring at different potentials with a low [Ru(bpy)_3_]^2+^ concentration. The negative ECL strategy was successfully applied also in different fields, paving the way to the research of different strategies for investigating the mechanism and exploiting new insights with the final goal to improve the sensitivity of the technique.

Electrochemiluminescence waveguide (ECLW) is another new technique with almost zero background and specific molecular functions, where single crystalline molecular wires of tris(1-phenylisoqsuinoline-C2,N)iridium(III) can act as a waveguide and ECL emitter.^41^ ECL can be generated at the interface between the wire and electrode surface (ITO) through the oxidation of both the wire and coreactant. The intensity is higher at the extremity, and the ECL generated can be propagated along the wire, although it is on a glass substrate and between wires next to each others. This behavior offers the possibility of contactless electrochemical analysis and study of biological samples with less or even without an electric disturbance.

The influence of an additional electric field can be useful for the higher performance of the ECL system [Ru(bpy)_3_]^2+^/TPrA. An enhanced electric field is produced at the heterogeneous interface between bowl-like microparticles and the ITO surface, thanks to the contact between two materials with different dielectric constants.^42^ The observable result is a ring-shaped emission due to the higher ECL intensity obtained at the border of microbowls correlated to the enhanced electric field.

The electric field accelerates electrochemical reactions, decreases the [Ru(bpy)_3_]^2+^, and increase the [Ru(bpy)_3_]^3+^ concentration at the heterointerfaces after the step potential application, enhancing the ECL intensity 4.3-fold with respect to the emission of gold microbowls and the ITO electrode. The reagents and ECL emission distribution were studied through COMSOL simulation, and it was in agreement with experimental results. The important role of the heterointerface electric field in promoting the electrochemical reaction and consequently enhancing the ECL emission was proved by decreasing the potential applied for ECL generation from 1.2 to 0.9 V where an ECL emission is still visible. The presence of an electric field not only accelerates reactions but also represents a different strategy to improve the electrochemical reaction efficiency. Finally, luminol was used instead of [Ru(bpy)_3_]^2+^ as a luminophore, but the enhancement of ECL intensity is only 2.5-fold due to limitations in the luminophore efficiency.

Luminol was used in two other ECL methods. The first one exploits a very high-density assay of wells of 20 nm surrounded by aluminum, which deplete ECL.^43^ The wells do not hinder the diffusion of luminol and hydrogen peroxide and may generate observable ECL spots. Furthermore, the wells can also be filled with glucose oxidase, which generates hydrogen peroxide in the presence of glucose, thus making the intensity measured at the wells dependent on glucose concentration. The second example exploits the defects of graphene oxide microsheets for visualizing the fluctuation of charge transfer resistance connected with the variation of the redox defect.^44^ Redox defects catalyze the electrochemical generation of oxygen radicals from water that increase the ECL emission from L012 (a type of luminol) and H_2_O_2_ and consequently its temporal resolution.

**Figure 2:**
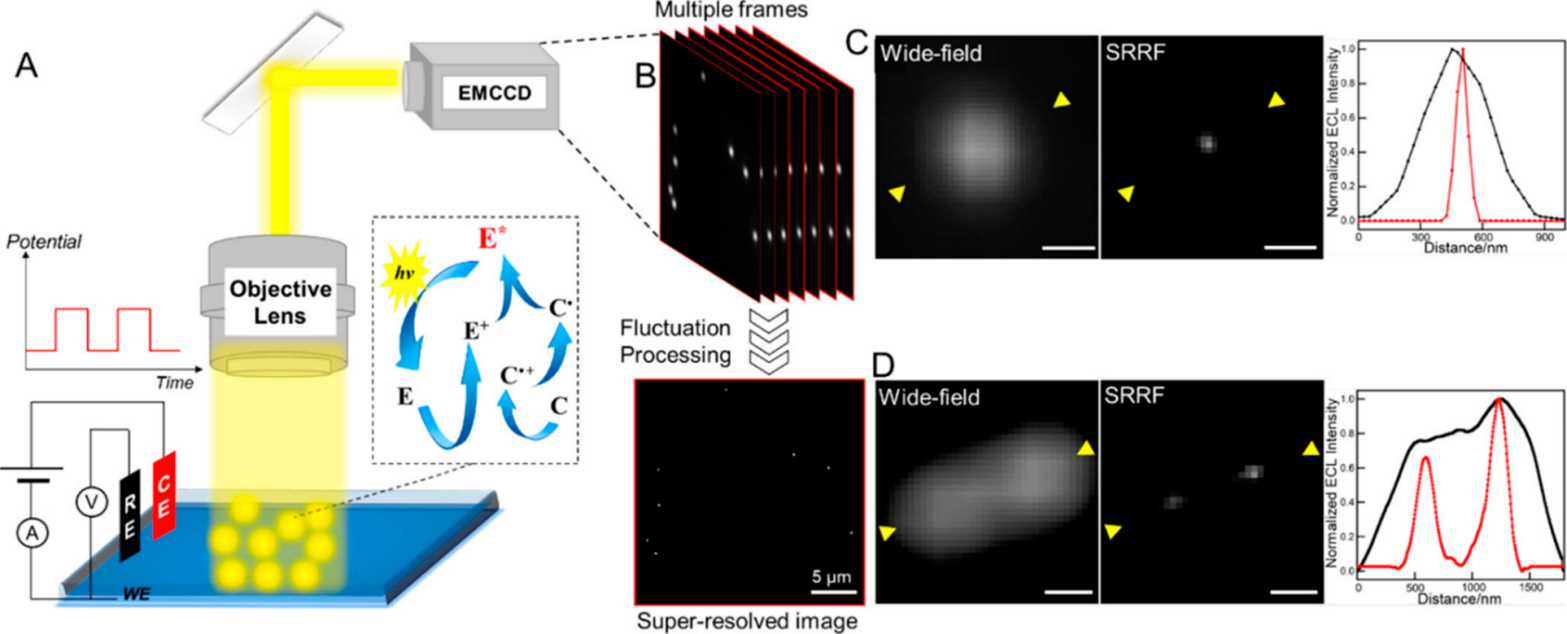
(A) Schematic illustration of the ECLM system for single-particle imaging. E and C represent the luminophore and coreactant, respectively, which are Ru(bpy)_3_^2+^ and TPrA. (B) Basic principle of SRRF analysis of multiple images. Wide-field and super-resolution ECL images of (C) a single Au nanosphere and (D) separated Au nanospheres and corresponding line profiles taken from the regions between the yellow arrowheads. Scale bar: 500 nm. Reproduced from Chen, M.-M.; Xu, C.-H.; Zhao, W.; Chen, H.; Xu, J. *J. Am. Chem. Soc.***2021**, *143*, 18511–18518 (ref (20). Copyright 2021 American Chemical Society.

A very recent work brings ECL microscopy to the limits of super-resolution, thanks to the combination with the super resolution radial fluctuations (SRRF) algorithm.^20^ The combination is possible because photons, in ECL, are generated in a stochastic way, and the temporal variation in consecutive ECL frames allows the image to be reconstructed by SRRF (setup and ECL images reported in Figure 2). ECLM could measure the facet- and defect-dependent activities of single nanoparticles, obtaining abundant details on electrocatalytic reactivities and their fluctuations within a single entity. A high spatiotemporal resolution was reached showing great potential in the field of energy materials and single-cell imaging and analysis.^20^ Finally, ECL microscopy can exploit the direct oxidative mechanism and the new insights discovered in the last years for the enhancement of ECL sensitivity and temporal and spatial resolutions. In order to increase and study the ECL generation, it is important to (i) modulate and study the TEL also through specific techniques like ECL self-interference spectroscopy;^45^ (ii) increase the lifetime of different ECL reagents; (iii) combine ECL with different strategies, materials, and geometries. The latter point is very useful for biosensoristic applications because it allows ECLM to reach super-resolution and the efficient visualization of the cells, subcellular organelles, and cell processes while avoiding their damage or death. Moreover, very recently Dong et al. developed a method that images the positions of single photons emitted by a single luminophore, where the reactivity of the coreactant and the dye were highly controlled. The application is very successful, and it gives a glimpse of a very bright future for ECL imaging.^6^

#### Applications of Homogeneous Mechanism

The ECL homogeneous mechanism possesses several advantages which enable its application to biomarkers detection and to the study of the behavior of various chemical and biological systems. This mechanism is highly represented within the ECL literature, and a section fully dedicated to its application is thus mandatory.

One interesting and useful field for sensoristic applications is forensic investigation, where fingerprint analysis has a prominent role. Some research studies were successfully carried out for the detection of fingerprints using ECL microscopy as well as for the recognition of exogenous substances on finger-marks. Recently, Li et al. have developed an “all-in-one-phone” device for fingerprints mapping and in situ biochemical sensing. They demonstrated the advantages of using a point-of-care device for the detection of exogenous substances in fingerprints: nicotine for instance enhances the ECL signal because it is a tertiary amine (like TPrA), while trinitrotoluene (TNT) quenched ECL because it is electron acceptor interfering with the ECL generation.^46^ Also the second- and third-level details of the fingerprints such as lakes, bifurcations, terminations, and crossovers are of great importance for personal identification. Starting from these assumptions, a method was developed for visualizing latent fingerprints by spatially selecting electropolymerization of luminol on an ITO electrode without using toxic or harmful reagents. The fingerprint deposited on the electrode acts as a mask, and the polyluminol is produced only on the bare electrode surface, resulting in a negative ECL fingerprints pattern.^47^

In a field like forensic analysis but more strictly correlated to security, Li et al. exploited the advantage of confined geometries and designed a silica nanoporous smartphone-based device for sensing nitroaromatic compounds, precursors for the manufacture of explosives in industry and military activities. This sensor shows high selectivity toward nitroaromatic compounds thanks to the enhanced ECL of nanopores modified with specific recognition polypeptides as largely explained in the previous section.^48^

**Figure 3:**
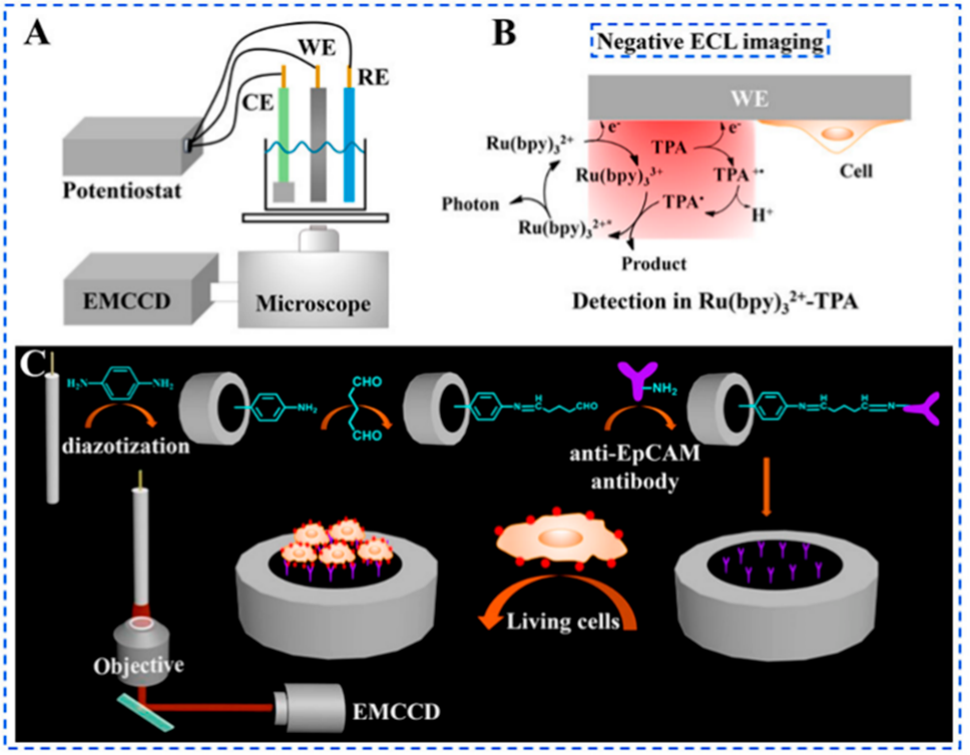
(A) Instrumental setup for the negative ECL imaging system [WE, glassy carbon electrode (GCE); CE, Pt foil electrode; RE, Ag/AgCl electrode (sat KCl)]; (B) schematic illustration of the negative ECL imaging method; and (C) schematic illustration of the construction of the sensing interface and the capturing of living cells for the morphological and quantitative analyses of living cells. Reproduced from Gao, H.; Han, W.; Qi, H.; Gao, Q.; Zhang, C. *Anal. Chem.***2020**, *92*, 8278–8284 (ref (49)). Copyright 2020 American Chemical Society.

Nanoconfined and nonconductive structures have previously been also used for cells visualization through the so-called “negative ECL” method, whose principle is shown in Figure 3B together with a quite common instrumental setup (Figure 3A).^7^ The same principle has been exploited to imagine MCF-7 living cells, immobilized on the glassy-carbon (GC) electrode through an antibody anti-EpCAM, as shown in Figure 3C. An electrical stimulation and H_2_O_2_ were used to stimulate cell and visualize their morphological modifications, in the presence of [Ru(bpy)_3_]^2+^ and TPrA.^49^

However, the direct ECL imaging is also very effective and was exploited to visualize cells without a hindrance effect on the electrode surface.^50,51^ These two studies required a film deposition on the electrode. In the first one, a permeable chitosan film was deposited on the FTO/TiO_2_/CS electrode prior to cell immobilization, and then a cell stimulating agent was added which triggered cells with H_2_O_2_ production. H_2_O_2_ is a reactive oxygen species (ROS) associated with the regulation of cellular processes *in vivo* and acts as a coreactant for ECL generation in the presence of luminol, allowing single-cell ECL imaging.^50^ In another work, a permeable and conductive graphene hydrogel layer was deposited on a GC electrode, without hindering K_2_S_2_O_8_ (coreactant) diffusion. K_2_S_2_O_8_ reacts with the glucose transporter 4 (GLUT4) expressed in human skeletal muscle cells and labeled with carbon dots, obtaining both the cell ECL image and the transporter quantification.^51^ These works are part of a consistent number of studies concerning the visualization of cellular modifications related to diseases with the aim to improve early diagnosis. In this field, single-cell ECL imaging plays an essential role in elucidating the biological mechanism of cell function with accurate detection of heterogeneity among cells. In order to address the diagnosis and treatment of tumors, gold nanocages (Au NCs) incorporating phorbol-12-myristate-13-acetate (PMA) were used to visualize HeLa cancer cells exploiting the DNA–RNA interaction. In this system, a DNA strand is conjugated with the Au NCs, and it selectively hybridizes with the miRNA-21 present in a cancer cell. The hybridization event causes a conformational change in the Au NCs and a subsequent release of PMA, which stimulates ROS production, including H_2_O_2_. ECL is generated by adding luminol in solution which reacts with H_2_O_2_ allowing one to detect miRNA-21 at the single cell level and to image the cell apoptosis process caused by the increased level of intracellular ROS (photothermal effect).^52^

Other homogeneous ECL imaging applications concern the study of the reaction mechanism and electrocatalytic activity of new micro- and nanosized materials. For example, electrocatalytic activity at different ZnO crystal facets was visualized and investigated via ECL imaging.^53^ Chen and co-workers discovered that the ZnO (002) facet is more energetically preferential than the ZnO (100) facet for O_2_ absorption through the oxygen reduction reaction, which catalyzes the luminol ECL emission at ambient conditions.

Ma et al. in their work describe a novel ECL blinking technique at the single-nanoparticle level to directly monitor the hydrogen evolution reaction (HER). They were able to visualize the growth and collapse of H_2_ nanobubbles released by carbon nitride nanospheres which acts as both ECL emitter and HER catalyst.^54^ Other researchers used [Ru(bpy)_3_]^2+^ and TPrA in solution to visualize the ECL emission on single 2D microsized gold nanocrystals underlying their catalytic effect.^55^ Instead, surface defects of reduced graphene oxide (rGO) microsheets adsorb luminol and H_2_O_2_, used as a luminophore and coreactant, respectively, allowing the evaluation of rGO electrocatalytic activity. The ECL emission is localized, and after oxygen plasma irradiation, more defects are visible and the emission is enhanced.^56^ Concerning materials and the role of oxygen, oxygen vacancies on the surface of rutile TiO_2_ nanoparticles were used for immobilizing H_2_O_2_, which upon electrochemical conversion to O_2_^*–^ and OH*, reacts with oxidized luminol to produce a continuous ECL light emission (steady-state luminescence). This emission is correlated with the concentration of hydrogen peroxide, leading to the local ECL visualization of hydrogen peroxide released by single cells.^57^ Innovative materials or common materials with novel features can be exploited by ECL microscopy for improving its performance, specifically in the biomedical field for the visualization of cells and the monitoring of released substances. An electrochemiluminescent nanocage array, composed of two layers of silica nanoporous membrane (SNM) with different porous sizes that trap ECL luminophore, was developed. Hence, an “on–off” ECL solid-state sensor was realized for detecting biomolecules, such as dopamine and hydrogen peroxide efflux from cells, without a direct contact with the living cell.^58^

Recently, ECL imaging was employed also for investigating boundaries and interfaces that play a key role in many chemical reactions. One example is the visualization of liquid/liquid interfaces exploiting the different solubility ratios between the coreactant sodium oxalate and solubility in water and the luminophore [Ru(dmbpy)_3_]^2+^ and solubility in an organic solvent. ECL can be generated only at the interface between water and solvent, obtaining the visualization of the interface and its thickness. Also, the gas/liquid interface is viewed in this work using an aqueous solution of oxalate and [Ru(bpy)_3_]^2+^. This was possible thanks to fine-tuning of the applied potential that activates water oxidation and creates an oxygen bubble, black with respect to the electrode, which enables the interfacial thickness measurement.^59^ In an another work, the same team described a method to quantify the entrapment of immiscible solvent in aqueous microdroplets through ECL microscopy. In this case, they used hydrophilic ECL reagents, [Ru(bpy)_3_]^2+^ and oxalate, to confine the ECL reaction in the aqueous phase, leaving the trapped organic solvent obscured.^60^ In this way, they were able to assess the actual contact area between the microdroplet and the electrode without using any geometry dependent modeling.

#### Catalytic Route Investigation

The ECL homogeneous mechanism includes a great number of applications that underlie the surface-confined nature of ECL imaging, and the chemical processes responsible for ECL signal generation are confined to the electrode surface.^3^ ECLM sensitivity has been successfully applied for the visualization of the cell and investigation of their processes, which is also thanks to the great improvement achieved from the application of a specific homogeneous ECL mechanism called the “catalytic route”. This pathway is the freely diffusing [Ru(bpy)_3_]^2+^ and more precisely the oxidized form [Ru(bpy)_3_]^3+^ that catalyzes the coreactant oxidation. Indeed in this mechanism, ECL generation is dependent on the [Ru(bpy)_3_]^3+^ lifetime and its reaction with the coreactant, while the coreactant radical cation lifetime is less important.^39^ This behavior is advantageous because the [Ru(bpy)_3_]^3+^ lifetime is usually much higher than the coreactant radical cation lifetime.^11^ Moreover, by simply modulating the concentration of oxidized luminophore, the ECL emitting layer can be increased/decreased from a few nanometers to several micrometers and is not limited to the first 3 μm,^36,39^ allowing one to spatially resolve objects at different distances from the electrode.

Ding et al. delved into the homogeneous mechanism responsible for ECL emission via the coreactant pathway.^61^ They combined the “negative ECL” microscopy strategy to explain the catalytic route for visualizing photoresist structures. Briefly, with [Ru(bpy)_3_]^2+^ and TPrA in solution, they observed a black spot corresponding to the photoresist structure instead of an emission and its dimension decreased with the increasing of the ECL emitting layer. This happens because a higher part of the spot is immersed in the ECL emission layer. This phenomenon can be tuned allowing the visualization of different biological photoresist structures, such as cell–matrix and cell–cell junctions, present, respectively, at very low and high distances from the electrode surface through a high bright-dark contrast and by tuning the ECL emission layer. Insight on the mechanism were obtained by modifying the concentration of [Ru(bpy)_3_]^2+^ and coreactant in solution. Decreasing the concentration of coreactant and increasing the [Ru(bpy)_3_]^2+^ one, the oxidized luminophore can diffuse freely and react with the coreactant far from the electrode increasing the emission layer.^61^ These results are very promising for future development of spatially selective microimaging in order to monitor the distribution of membrane proteins and their movement or process at the cellular and subcellular levels.

Moreover, the catalytic route allows the direct visualization of the entire polystyrene bead with a 10 μm diameter and the upper cell membrane.^62^ In this case, the ECL emission comes from reaction between [Ru(bpy)_3_]^2+^ free in solution and nitrogen-doped carbon dots (NCDs), used as coreactants, that were attached to the microbeads through an amide bond. Thanks to the increase of the ECL emitting layer and the change of focal planes, all the beads can emit and their ECL profile can be reconstructed. NCDs can also be coupled with PSBP peptide allowing the recognition of phosphatidylserine (PS) externalized after apoptosis of HeLa cells.^62^ In this way it is possible to distinguish the normal cells from the apoptotic ones with huge sensitivity.

**Figure 4:**
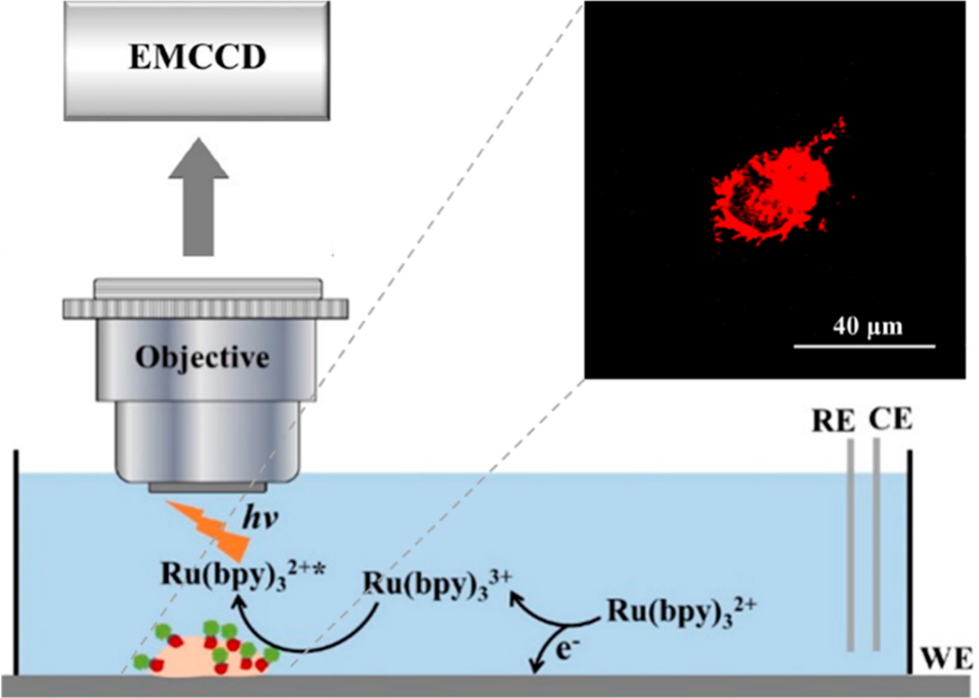
ECL imaging of the carcinoembryonic antigen (CEA) on cell membranes was shown on the top-right and was detected with a microscope coupled to an EMCCD camera. Cell visualization depends on the catalytic route of ECL reactions between freely diffusing [Ru(bpy)_3_]^2+^ and Hi-AuNF@G-ssDNA-Apt coreactants. Reproduced from Chen, Y.; Gou, X.; Ma, C.; Jiang, D.; Zhu, J.-J. *Anal. Chem.***2021**, *93*, 7682–7689 (ref (63)). Copyright 2021 American Chemical Society.

The main improvements were further reached by the use of nontoxic coreactants and by the use of the z-scan of the ECL emitting layer at the cell. Concerning the coreactant, Chen et al. presented another very interesting and efficient synergistic coreactant with good stability and negligible cytotoxicity: a guanine-rich single-stranded DNA (G-ssDNA)-loaded high-index faceted gold nanoflower (Hi-AuNF).^63^ HiAuNF is a very efficient electrocatalyst that when combined with G-ssDNA, the coreactant, allows the enhancement of ECL microscopy efficiency, catalyzing the oxidation of the luminophore free in solution [Ru(bpy)_3_]^2+^. The visualization of the cell is possible thanks to the ability of this material to selectively recognize biomarkers and to be catalytically oxidized by [Ru(bpy)_3_]^3+^ at a long distance from the electrode (Figure 4).^63^

However, the coreactants can directly and naturally come from the internal part of cells, allowing the visualization of intracellular structures and dynamic transport without the need for labeling.^64^ [Ru(bpy)_3_]^2+^ is used as a molecular antenna and optical readout, reacting with an internal amine-rich biomolecule that acts as a coreactant. [Ru(bpy)_3_]^3+^ enters into the cell for visualization of the intracellular structures of the nucleus, nucleolus, and rough endoplasmic reticulum, visualizing an increasing intensity due to different quantities of the amine-rich structures, like RNA and DNA. Moreover, some important cellular processes can be revealed. The migration of [Ru(bpy)_3_]^3+^ into the cells can be monitored: (i) it is very fast if the membrane is permeabilized; and (ii) it starts after 6 s applying a constant 1.3 V. In this last case, the lipid bilayer membrane barrier is electroporated or electropermeabilized when the transmembrane potential reaches a critical value (0.5–1.5 V). These pores allow the passage of [Ru(bpy)_3_]^3+^, connecting the intracellular environment with the extracellular one and giving access to the intracellular structure and dynamic processes, also through the observation of electroporation rate and [Ru(bpy)_3_]^2+^ velocity of diffusion.^64^

All the visualization of intracellular structures, cells, cell junctions, and microbeads is possible due to the long lifetime of [Ru(bpy)_3_]^3+^ with respect to very short TPrA and HEPES-associated radicals.^65^ The catalytic route also represents a very promising direction to follow for the sensitivity enhancement of ECL imaging and consequently the development of ECL microscopy based biosensors.

### Bipolar Electrochemistry-Electrochemiluminescence Microscopy

An important field is represented by bipolar electrochemistry-ECL (BPE-ECL) microscopy that is a very useful technique, in particular, in biological fields because it allows generation of ECL without the need of direct contact between the emitting sample and the electrode.^66,67^ BPE is based on the simple application of an electric field across an electrolyte solution, and its advantages are the control provided by the field strength and direction and its versatility because any kind of conducting object can theoretically be polarized in this wireless mode. Sojic and co-workers made important breakthroughs combining the BPE and ECL microscopy with both magnetic and electric fields, tracking the light emission of rotating iron wire coated with gold in a solution of Ru(bpy)_3_^2+^ and TPrA.^68^ In their work, Ru(bpy)_3_^2+^ and TPrA are oxidized at the anode and ECL is turned on and off consistently when the longitudinal axis of the wire approaches the position aligned with the electric field, resulting in an ECL intensity proportional with the magnetic field. However, an ECL decrease is observed for a higher magnetic field due to water oxidation which alters the local pH, disturbing the TPrA oxidation at the electrode. ECL emission can be enhanced by the convection resulting from the rotational motion of the wire: the mass transport is constant and does not limit the ECL generation.

ECL imaging has also found many other applications using bipolar electrodes (BPE). An example of a simple closed bipolar electrodes-based ECL (BPEs-ECL) imaging strategy was developed utilizing functional nanoprobes of heterogeneous Ru(bpy)_3_^2+^@SiO_2_/Au nanoparticles for a visual immunoassay of prostate specific antigen (PSA) in the serum samples and on the surface of an individual cancer cell. The synergic amplifying effect and the consequently introduced multiple-assisted ECL signal amplification allows a sensitivity increase of ECL microscopy, and the detection of tumor markers is expressed on a single cell and not only in body fluids.^69^

The special features of BPE helped in the detection and quantification of H_2_O_2_ or glucose within living cells. The researchers used a pipet tip, on whose walls a porous structured Pt coating was deposited and able to adsorb molecules, such as luminol; because of the combination between its very narrow area and lower voltages than in the common BPE, the cell life was highly preserved.^70^ Although the applications on the cell study are the most studied and promising, BPE have also been tested for other interesting applications. Ismail and co-workers combined ECL and BPE for monitoring the evolution of the surface reactivity of silicon-PDMS microfluidic chips, an ECL imaging approach that opens exciting perspectives for the precise understanding and implementation of electrochemical functionalization on passivating materials.^71^ Instead, looking at the importance of food quality, a bead-based immunoassay sensor was developed for detection of *Salmonella typhimurium*, taking advantage of the BPE electrode and both Ir and Ru complexes. From tests carried out on milk, meat, and sweets, at different concentrations of analyte, they have obtained different colors of emission visible to the naked eye.^72^

Another important application of BPE required the fabrication of massive arrays of carbon bipolar ultramicroelectrode. Such an approach was used, for instance, for imaging the pressure-driven flow of redox species from a micropipet. Once again, confined geometries are very useful, as depicted in previous paragraphs. UME arrays are suitable for ECL microscopy imaging of fast and dynamic redox processes, with the resolution at a single electrode and imaging of variable redox concentrations.^73^

Finally, BPE-ECL and more generally light emission generated by TPrA and Ru with glassy carbon beads exposed to an electric field can be used for creation of innovative devices.^74^ For example, numerous micro/nano-BPEs (e.g., carbon microbeads, multiwalled carbon nanotubes) were dispersed in solution, and agarose gel was employed to keep them well separated during ECL analysis. Thanks to the simultaneous wireless bipolar addressing of these BPEs, intense 3D ECL in the entire solution was generated instead of 2D ECL confined to the electrode surface.^75^ This device was also able to image spatial variations of the concentration and composition of inhomogeneous samples, another great step toward super-resolved ECL microscopy.

### Heterogeneous Mechanism for Temporal and Spatially Resolved ECLM

A heterogeneous mechanism, as explained before, is responsible for ECL generation when the luminophore is constrained far from the electrode and unable to diffuse. In fact, in most analytical methods, the luminophore is attached to a sensing element and ECL emission is triggered exclusively by the radicals obtained by the anodic oxidation of TPrA.

The ECL immunoassay represents one of the principal methods for sensitive and accurate detection of diagnostic markers or biomarkers for the prediction of diseases and it is at the basis of commercial devices like Elecsys widely used in biological and clinical fields.^3,5^ Elecsys is a beads-based immunosystem, where a biotinylated sandwich immunoassay, labeled with luminophore [Ru(bpy)_3_]^2+^, is attached to a micromagnetic bead through the strong biotin–streptavidin bond. Beads are attracted toward the electrode surface by a magnet and, after TPrA addition and potential application, ECL emission is detected. The beads-based immunoassay system is governed by the ECL heterogeneous mechanism. [Ru(bpy)_3_]^2+^ is attached to the beads and cannot be oxidized by the electrode because it is too far from it (Figure 1b). The heterogeneous mechanism, proposed by Miao et al.^11^ at the beginning of the 21st century, has consistently been investigated since its introduction representing the main mechanistic working hypothesis for the interpretation of experimental results in a vast variety of different molecular, micro, and nanosystems. Such a mechanism was successfully applied in the ECL microscopy field in order to deeply study the emission process and improve its applications especially in biosensoristics, taking advantage of the higher spatial and temporal resolutions with respect to the homogeneous mechanism, also paving the way to the development of super-resolution advancements. The need of labeling the sensing element makes the heterogeneous mechanism the most applied in the biological field. Chen et al. took into account the label capacity of luminophores and identified the most suitable luminophores through DFT theoretical calculations and the correlation between redox potential and emission.^76^

ECL heterogeneous mechanism is mainly triggered by TPrA oxidization at the electrode, and its radical lifetime is the main limiting step. This behavior has been studied by Sentic et al. in 2014 using top or side view optical images of beads functionalized with [Ru(bpy)_3_]^2+^ and by calculating the ECL emission layer thickness and its dependence on TPrA^•+^ lifetime.^39^ The ECL emission was higher in the first 500 nm, and the total emission layer thickness was around 3 μm, with the optical observation supported by theoretical simulation. The thickness of the emission layer is limited by the short lifetime of TPrA^•+^ (200 μs). The influence of the ECL emission layer and coreactant diffusion in ECL imaging was described by Valenti et al. in the visualization of tumor cells thicker than 3 μm.^65^ Here, luminophores were functionalized with antibodies specifically recognizing antigens overexpressed onto the cell membrane. The authors were able to visualize ECL emission only from the cell borders because the luminophores in the central part of the cell are too far from the electrode to be reached by the short-lived coreactant radicals, and this area cannot therefore contribute to the overall emission.^65^ Voci et al. overcame this limitation adopting a permeabilization protocol for cells, thus allowing the coreactant to pass through the cell and be oxidized at the electrode to create the luminophore excited state throughout all the cell.^77^

ECLM is intrinsically a surface-confined technique; thus, the luminophore location on the electrode is a crucial point to consider. With the aim to achieve higher ECL signals using the heterogeneous mechanism, dye-doped silica nanoparticles (DDSN) were demonstrated to be a useful strategy to enhance the signal of built sensors.^21,78,79^ The main issue related to the use of nanoparticles (NPs) is that coreactant radicals generated at the electrode surface have to diffuse and interact with luminophores inside the NPs within their lifetime length. To overcome this problem, researchers acted on either the synthetic procedure or the coreactant choice.^80,81^ In particular, streptavidin coated micromagnetic beads labeled with biotinylated [Ru(bpy)_3_]^2+^-doped silica nanoparticles showed an ECL signal intensity up to 660-fold higher than beads labeled with single [Ru(bpy)_3_]^2+^ luminophores, which is the analytical approach adopted in commercial ECL-based immunoassay systems.^5,82^ The ECL intensity increase was mostly associated with the presence of a larger number of [Ru(bpy)_3_]^2+^ complexes with additional positive effects on the ECL signal stability due to the presence of the silica matrix.^82^ An application example of nanomaterials that concentrate a huge number of luminophores is represented by [Ru(bpy)_3_]^2+^-doped silica/Au nanoparticles. They were attached to the antibody that recognizes a specific protein as ECL labels and allows one to image a single biomolecule and a cellular membrane protein.^83^

**Figure 5:**
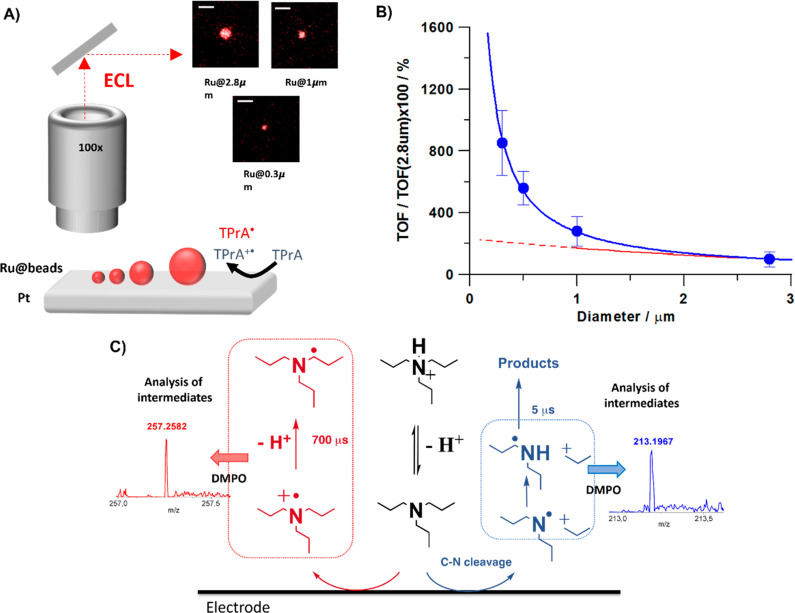
(A) Schematic representation of the surface generation-bead emission experiment on beads with different sizes where Ru@2.8 μm, Ru@1 μm, and Ru@0.3 μm are ECL images of magnetic beads labeled with [Ru(bpy)_3_]^2+^ with a diameter of 2.8, 1, and 0.3 μm, respectively. Magnification ×100; scale bar 5 μm; potential applied, 1.4 V (vs Ag/AgCl, 3 M KCl); acquisition time, 0.5 s. (B) Turnover frequency (TOF) as a function of bead size. (C) Schematic representation of the proposed parallel pathways for the tri-*n*-propylamine (TPrA) oxidation at the electrode where TPrA^•+^ and TPrA^•^ are generated (red pathway) and where dipropylamine radical (DPrA) is generated (blue pathway). The scheme reaction is supported by spin-trapping experiments with 5,5-dimethyl-pyrroline *N*-oxide (DMPO), which stabilized the radicals and allowed identification by mass spectrometry analysis (MS) and electron paramagnetic resonance (EPR). The inset shows the MS analysis for the possible adducts DMPO-TPrA and DMPO-DPrA. Reprinted by permission from Springer Nature Limited: Nature Communications, Zanut, A.; Fiorani, A.; Canola, S.; Saito, T.; Ziebart, N.; Rapino, S.; Rebeccani, S.; Barbon, A.; Irie, T.; Josel, H.-P.; et al. Insights into the Mechanism of Coreactant Electrochemiluminescence Facilitating Enhanced Bioanalytical Performance. *Nat. Commun.***2020**, *11* (1), 2668, DOI: 10.1038/s41467-020-16476-2 (ref (4)). Copyright 2020 Springer Nature Limited.

Other factors contribute to the ECL signal decay, for example, the oxidation of the working electrode provokes a decrease in the oxidation rate of TPrA. Dutta et al. managed to reduce such detrimental factors applying a cathodic surface treatment to the working electrode. They used nonconductive polystyrene (PS) beads with the [Ru(bpy)_3_]^2+^ complex attached via a sandwich immunoassay or a peptide bond, and the ECL emission was observed at a GC electrode.^84^ The ECL emission time profile was monitored after 1.1 V application. The anodic pulse may induce modifications of the working electrode, depending on the material.^22^ In the case of a GC electrode, oxygen-containing surface species are formed which may decrease the efficiency of TPrA oxidation also scavenging TPrA^•+^ with a consequent decrease of ECL efficiency. A cathodic treatment may restore the electrode pristine properties thus regenerating the initial ECL intensity.^84,85^ For example, in the case of cells attached to a GC electrode, several pulses of −1.8 V potential with 1 s duration were applied without generating hydrogen or damaging cells.^85^ This treatment avoids the strong decrease of ECL signal observed without treatment by creating a more hydrophobic surface with consequent faster kinetics of TPrA oxidation and removing the adsorbed electrogenerated species. The oxidation of TPrA is the most important step in the heterogeneous mechanism, and our group introduced the concept of its tuning for reaching an improvement in the sensitivity of the technique: a new path at short distance was discovered and the introduction of additive successfully optimized coreactant oxidation, obtaining a higher ECL signal.^4^ Recently, Bard and co-workers’ heterogeneous ECL mechanism^11^ was revisited highlighting the presence of a supplementary new mechanistic pathway, operating at very short distances (<1 μm) from the electrode surface, previously unobserved. The study relied on the combination of two complementary approaches to investigate the spatial distribution of ECL emission efficiency, namely, (i) a platinum hemispherical microelectrode (where TPrA is oxidized) positioned at controlled distances (0.1–2.8 μm) from a transparent electrode bearing a [Ru(bpy)_3_]^2+^ monoloayer and (ii) the use of micromagnetic beads labeled with [Ru(bpy)_3_]^2+^ with diameters ranging from 0.3 to 2.8 μm. As expected, on the basis of the heterogeneous mechanism, the decrease of the tip–electrode distance or bead dimensions brought about an increase of ECL emission explained, in both cases, by the fact that the short-lived TPrA^•+^ would be intercepted by luminophores more efficiently. Quite unexpectedly, however, the experiments evidenced the presence of an additional mechanistic pathway associated with a new species with a much shorter lifetime than TPrA^•+^ (200 μs) and responsible for a huge increase of ECL efficiency at distances <1 μm (Figure 5A,B). This was attributed to a parallel mechanism of TPrA oxidation where a N-centered dipropylamine radical is generated by C–N bond cleavage concerted with TPrA oxidation (Figure 5C). Exploiting these findings, a new branched amine coreactant (i.e., *N*-dipropyl isobutyl amine, DPIBA) was proposed, which features a more stable carbocation forming from the C–N bond breaking. DPIBA was mixed with TPrA in experiments aimed to maintain high the efficiency at a long distance (with TPrA) and enhance at the same time the contribution at short distances according to the new mechanism (with DPIBA). A significant increase in ECL intensity and sensitivity was in fact observed in a series of Elecsys assays in the Roche Diagnostics Cobas e 801 immunoassay analyzer.^33^

**Figure 6:**
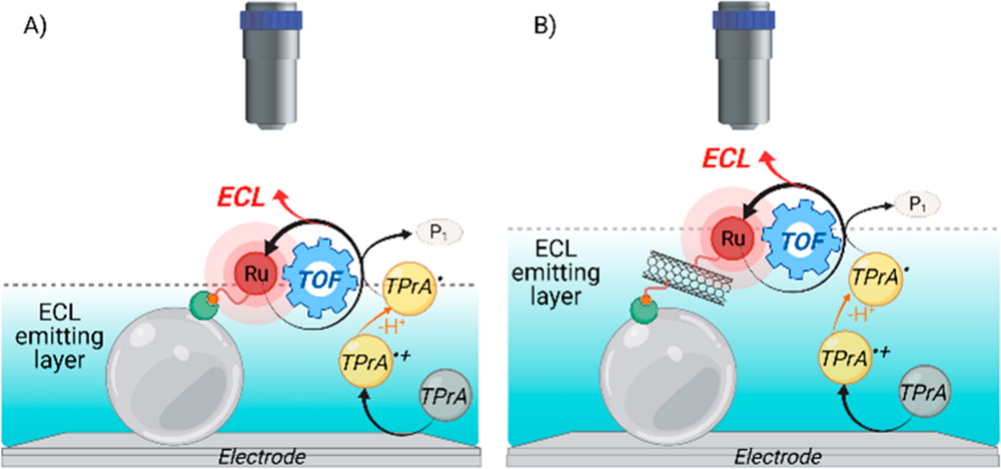
“Oxidative-reduction” coreactant mechanisms for the electrochemiluminescence emission of micromagnetic bead (light gray sphere) systems (biotin, red circle, and streptavidin, green shape), involving TPrA as the coreactant and [Ru(bpy)_3_]^2+^ as the luminophore. (A) Only TPrA is oxidized on the electrode. (B) [Ru(bpy)_3_]^2+^ is also oxidized on the double-walled carbon nanotubes (f-CNTs). Reproduced from Rebeccani, S.; Wetzl, C.; Zamolo, V. A.; Criado, A.; Valenti, G.; Paolucci, F.; Prato, M. *Chem. Commun.***2021**, *57*, 9672–9675 (ref (91)), with permission of The Royal Society of Chemistry.

The coreactant radical cation lifetime can in principle be extended/reduced acting on the reaction medium. This novel concept was recently investigated by using phosphate buffer solutions with different strengths.^86^ Changes of PB concentration can in fact modulate the TPrA deprotonation rate: at higher concentrations, phosphate ions are more readily available for buffering the hydrogen ions released from TPrA^•+^, making its decay faster. The TPrA^•+^ lifetime decrease shortened the TPrA^•+^ diffusion length. As with a chemical lens, the result is a thinner emission profile confined to the electrode surface and associated with a lower ECL intensity because a smaller area of the microbeads is reached by TPrA^•+^.^86^ Changes in the ECL emission layer thickness can also be obtained by exploiting the electrical properties of carbon nanomaterials.^23,87^ The ECL emission layer modulation was just tested through an additional conductive layer around the sample formed by graphene oxide and 2D conductive material.^88−90^ However, double-walled carbon nanotubes (f-CNTs) labeled with [Ru(bpy)_3_]^2+^ complex were used first for the functionalization of micromagnetic beads.^91^ Because of their intrinsic conductivity, the nanotubes form a conductive network all around the beads which activate an additional mechanism for ECL generation which is usually not active in the heterogeneous beads system. The luminophore is in fact in close contact with a conductive material, independent from its distance from the underlying electrode, and it can be oxidized together with the coreactant, thus making the homogeneous ECL path viable (see Figure 6). The concomitant presence of the new mechanism and the heterogeneous one, ECL emission layer and ECL intensity are enhanced with respect to the conventional micromagnetic beads labeled with [Ru(bpy)_3_]^2+^ complex without f-CNTs. All these findings open new and very promising routes toward an increase in the sensitivity of ECL immunoassays based on the ECL imaging technique. It has been highlighted that not only the strategies are very important, like the change in PB concentration, but also the choice of types of coreactants and luminophores. Moreover, two other very interesting research studies exploited ECL imaging for building an intramolecular system and for visualizing the cell process.

The cellular death (apoptosis) diagnosis will contribute to evaluate the occurrence of diseases and the therapeutic effect of antitumor pharmaceuticals. The apoptosis of cells usually induces a decrease in the expression of epidermal growth factor receptor (EGFR) and promotes phosphatidylserine (PS) eversion on the cell membrane. In this context, Liu and co-workers developed two cell@probes (g-C3N4-PSBP and Au@L012@EGF) capable of identifying EGFR and PS, respectively, showing two well-separated ECL signals during a potential scanning on a modified electrode.^92^

Wang et al.^93^ developed a coreactant-embedded ECL microimaging system where amine conjugated polymer dots are used as a unique system instead of the one with the luminophore and coreactant separated. This strategy showed a strong ECL emission due to very efficient and fast intramolecular electron transfer together with the innovative conjugated structure.

## Conclusions

In the last 20 years, ECL has been widely adopted as a powerful analytical technique because an ultrasensitive optical signal can be acquired after an electrochemical trigger, which lowers the background signal with respect to photoluminescence analysis with a unique signal-to-noise ratio. In the last 2 years, ECL has received even greater interest as a powerful analytical imaging technique and has been successfully exploited to visualize microobjects and entities, also through studies on the ECL mechanism generation. Mechanistic investigations allow one to single out different strategies for increasing signal intensity and temporal and spatial resolutions in the technique (see Table 1 for some resuming points) without damaging biological objects in the case of a biosensor for early disease diagnosis and clinical monitoring. Every year, two billion ECL based immunoassays are run worldwide and recent developments gave an important boost to the technique improvement toward single-protein-molecule imaging and single-molecule detection and, consequently, to the application of ECLM as a super-resolved technique.

**Table 1:** Summary of All the Mechanism Illustrated with Relative Limiting Factors, Advantages, and Strategies for ECLM Signal Enhancement^a^

type of mechanism	main limiting factors	advantages	strategies for ECL higher sensitivity
homogeneous	τ of C radical cation and D ox form	• absence of labeling	• confined geometries
			• two materials with different dielectric constant in contact
			• image eloborations with SRRF algorithm
catalytic	τ of D ox form	• absence of labeling	• increase D concentration and acquisition time
		• higher TEL	• different type of C
BPE-ECL	τ of C radical cation and D ox form	• absence of contact between sample and electrode	• combination with magnetic fields
			• nanostructures
heterogeneous	τ of C radical cation	• higher spatial and temporal resolution	• different D for optimization of C diffusion toward D
		• simulation of real immunoassay application	• two different mechanisms at short and long distances
			• increase of TEL (chemical lens, conductive materials)
			• electron cathodic treatment

a C is for coreactant; D is for dye/luminophore.
